# Lower Limit of Normality of Segmental Multilayer Longitudinal Strain in Healthy Adult Subjects

**DOI:** 10.3390/jcdd11040102

**Published:** 2024-03-28

**Authors:** Liviu Moraru, Oana Mirea, Despina Toader, Mihaela Berceanu, Sorina Soldea, Alexandru Munteanu, Ionuț Donoiu, Victor Raicea

**Affiliations:** 1 Department of Anatomy, UMFST, 540142 Targu-Mures, Romania; dr.liviu.moraru@gmail.com; 2 Department of CardioVascular Surgery, IUBCVT, 540142 Targu-Mures, Romania; 3 Department of Cardiology, Emergency County Hospital Craiova, 200638 Craiova, Romania; despinamtoader@yahoo.com (D.T.); sorina.iordache93@gmail.com (S.S.); ionut.donoiu@umfcv.ro (I.D.); 4 Department of CardioVascular Surgery, Emergency County Hospital Craiova, 200638 Craiova, Romania; mihaela.florescu0222@yahoo.com (M.B.); dr.raicea.victor@gmail.com (V.R.); 5 Department of General Surgery, Emergency County Hospital Craiova, 200638 Craiova, Romania; alexandru.munteanu@umfcv.ro

**Keywords:** speckle tracking echocardiography, segmental strain, subendocardial strain, subepicardial strain, mid-myocardial strain

## Abstract

Speckle tracking echocardiography is an advanced imaging technique that allows for a more detailed assessment of cardiac global and regional function. Reference values for segmental longitudinal layered strain (subendocardial, mid-myocardial, and subepicardial) are scarce, limiting the clinical use of these measurements in clinical practice. Two hundred consecutive Caucasian healthy subjects (mean age = 37 ± 11 years) were enrolled in the study. The mean values of global longitudinal strain (GLS) for endocardial (Endo), mid-myocardial (Myo) and epicardial (Epi) layers were −22.9 ± 2.7, −20.0 ± 2.4 and −17.5 ± 2.1, respectively. The GLSEndo/GLSMyo ratio was 1.1 ± 0.05, while the GLSEndo/GLSEpi ratio was 1.3 ± 0.05. The apical strain-sparing ratio was >1 in 10% of the subjects (endocardium) and 7% (mid-myocardium). The lower limits for segmental LS were as follows: for endocardial LS, −10% (basal), −12% (mid), −14% (apical); for mid-myocardial LS, −10% −10% (basal), −10% (mid), −10% (apical); and for epicardial LS, −7% (basal), −8% (mid), −8% (apical). The findings of this study provide data regarding the lower limit of normality of LS for each LV segment and suggest, for practical considerations, that an LS value below 10% should be considered abnormal in any segment. Further larger studies are warranted to confirm these findings.

## 1. Introduction

Speckle tracking echocardiography (STE) is currently recommended as an imaging tool to evaluate left ventricular (LV) function [1]. An expert consensus to guide image acquisition, postprocessing and interpretation is currently available [1]. Moreover, global longitudinal strain (GLS) has gained importance in clinical practice, particularly in conditions where early detection of LV dysfunction is crucial.

Considering the incremental diagnostic and prognostic value of GLS in a broad spectrum of cardiac diseases [2], numerous attempts have been made to identify whether regional strain patterns can hold any clinical value. Preserved longitudinal deformation in the LV apical segments (apical sparing) suggests the etiology of amyloidosis in hypertrophic hearts [3], while the presence of early septal stretch combined with late lateral wall contraction in subjects referred for cardiac resynchronization therapy predicted response to the therapy [4]. Moreover, the induction of acute coronary ischemia was followed by immediate changes in regional myocardial deformation [5]. Last, regional myocardial dysfunction assessed with STE proved to correlate with the presence of fibrosis assessed with cardiac magnetic resonance (CMR) and showed prognostic value in subjects with acute myocarditis [6,7]. These reports clearly outline the potential implications of segmental strain in clinical practice.

However, the widespread clinical use of segmental strain measurements is hampered by the relatively poor reported reproducibility, the inconsistency of tracking algorithms between different vendors and the absence of well-established normal ranges for strain values in different myocardial layers and segments [8]. This makes it challenging for clinicians to interpret the significance of abnormal findings.

In this study, we aimed to provide normal ranges for segmental layered longitudinal strain (LS) in a cohort of subjects without history or evidence of cardiovascular disease using a commercially available postprocessing software. Providing ranges of normality for segmental strain values has clinical implications, facilitating the differentiating between “normal” and “pathologic” values of segmental strain and highlighting potential underlying pathology.

## 2. Materials and Methods

Two hundred healthy young subjects (mean age 37 ± 11) were prospectively enrolled in the department of Cardiology of the County Hospital of Craiova. The participants were aged from 18 to 55 and revealed no history of cardiovascular disease during anamnesis; they were either collected from the community or underwent echocardiographic examination due to various symptoms (palpitations, atypical chest pain) or reasons (preoperative assessment, routine check-up). We included consecutive subjects that met the following criteria: (a) age > 18 years old and (b) no documented history of cardiovascular diseases such as hypertension, coronary/peripheral arterial disease or diabetes mellitus. 

Blood pressure, heart rate and anthropometric information were collected at the time of study, including age, weight, body surface area (BSA) and body mass index (BMI).

The study was approved by the hospitals’ ethics committee, and informed written consent was obtained from all subjects.

### 2.1. Two-Dimensional Echocardiographic Imaging and Measurements

Echocardiographic images were obtained using a Vivid E9 ultrasound system (GE Vingmed Ultrasound, Horten, Norway). We recorded all echocardiographic measurements in accordance with the recommendations for chamber quantification [9]. Subjects with poor image quality (>3 segments with artifacts, shadows, etc.) were not included in the study.

Left ventricular apical 4-, 3-, 2-chamber views were acquired with a frame rate above 60 fps. For LV function, biplane volumes were obtained from the apical 2- and 4 chamber views, and ejection fraction (modified Simpson’s rule) was used to assess LV systolic function. Devereux’s formula was used to calculate LV mass [9].

Diastolic function was assessed from the mitral early (E wave) and late diastolic (A wave) flows obtained from the PW spectral envelope. The ratio E/e’ was used to estimate LV filling pressures.

Left atrium (LA) volume was assessed from modified 4-chamber view. RV end-systolic area (RV-ESA) and end-diastolic area (RV-EDA) were assessed by tracing the endocardium line with the fractional area shortening (FAS) calculated as the difference in per cent between the EDA and ESA. Tricuspid annular plane systolic excursion (TAPSE) was measured from the apical four chamber view as an estimate for RV function. Parameters were indexed to BSA if suitable.

### 2.2. Two-Dimensional Echocardiographic Strain Measurements

**Figure 1:**
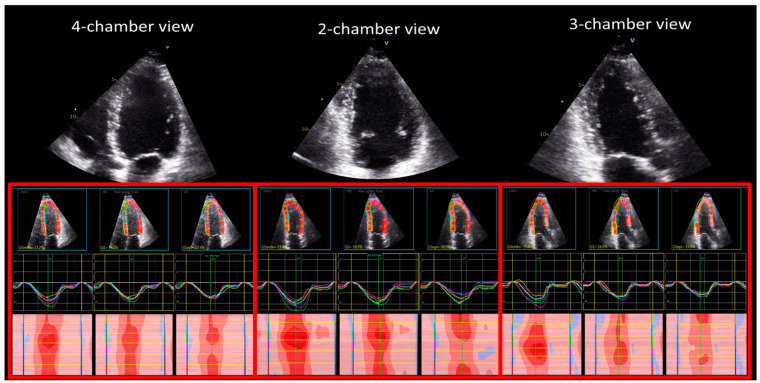
Measurement of layered longitudinal strain from the 4-chamber, 2-chamber and 3-chamber views.

The digital examinations were processed offline by a single experienced reader (OM) using EchoPac version BT204 dedicated software. Global longitudinal strain (GLS) for the endocardial (GLSEndo), mid-myocardial (GLSMyo), and epicardial (GLSEpi) layers were measured from the apical projections (Figure 1). The analysis was performed by manually tracking the endocardium line. The range of interest was then adjusted consistent to myocardial thickness. The evaluation or regional analysis was conducted through visual inspection and segments were excluded in case of suboptimal tracking (non-distinguishable speckles, presence of reverberations or sliding of the segment).

Segmental longitudinal strain was analyzed using a predefined 18-segment model of the LV, i.e., three segments per wall (Figure 1), as recommended by current consensus papers [1].

The relative apical sparing pattern (RASP) was quantified for all layers using the formula (average apical strain)/[(average basal strain) + (average midventricular strain)] [3].

For all strain measurements, aortic valve closure (AVC), mitral valve opening and mitral valve closure were manually calculated using the pulsed wave Doppler obtained by sampling LV outflow tract and mitral diastolic flow, respectively.

To assess the interobserver reproducibility of the measurements, a second analysis on a subset of 20 subjects randomly selected was performed by the examiner at approximatively 2 weeks after the initial reading, blinded to the results.

### 2.3. Statistical Analysis

Clinical and echocardiographic characteristics are provided as mean ± standard deviation for continuous variables, or as absolute number or percentage for categorical variables. Variables were checked to be normally distributed and to have equal variances. To compare data between the predefined study groups, the unpaired *t*-test was used.

Linear regression analysis was used to evaluate the independent demographic and echocardiographic predictors of layered GLS.

Intra-observer variability of multilayer strain was assessed by calculating intra-class correlation coefficient (ICC) and 95% confidence intervals (CIs) of the strain components. The result was interpreted as excellent correlation when ICC above 0.75 [10]. Next, for segmental strain, we calculated the mean difference of absolute values.

Statistical analyses were performed using SPSS version 17.0 (SPSS Inc., Chicago, IL, USA). Significance was set at a two-tailed probability level of <0.05.

## 3. Results

### 3.1. Demographic and Standard Echocardiographic Characteristics

We included 200 healthy volunteers (males = 130). A summary of the patient’s demographic and standard echocardiographic measurements is provided in Table 1. The mean systolic and diastolic blood pressure were 118 ± 9 mm Hg and 68 ± 7 mm Hg, respectively. The average resting heart rate was 81 ± 14 bpm.

All subjects demonstrated normal LV ejection fraction (mean 59 ± 5%, range between 50 and 74%) and normal LV indexed mass. According to the current recommendations [11], diastolic function was normal in 90% of the subjects. The remaining subjects presented with impaired relaxation. All subjects showed normal filling pressures (average e/e’ = 5 ± 1). The mean LAVI was 21.9 ± 5.7 mL/m^2^.

Right ventricular function was normal as assessed with TAPSE (22 ± 3 mm) and FAS (45 ± 9%).

There was no significant difference in age, but females had lower BSA and BMI, lower LV wall thickness and mass (Table 2).

### 3.2. Global Longitudinal Strain

Mean frame rate was 65 ± 10 frames per second. In all subjects, an endocardial (highest) to epicardial (lowest) gradient was observed (*p* < 0.01).

Average GLS was −22.9 ± 2.7%, −20.0 ± 2.4% and −17.5 ± 2.1% for endocardial, mid-myocardial and epicardial, respectively. The GLSEndo/GLSMyo ratio was 1.1 ± 0.1, while the GLSEndo/GLSEpi ratio was 1.3 ± 0.1.

Women showed significantly higher values for GLSEndo (−23.6 ± 2.6% vs. −22.4 ± 2.8%, *p* < 0.01), GLSMyo (−20.7 ± 2.3% vs. −19.6 ± 2.4%, *p* < 0.01) and GLSEpi (−18.2 ± 2.2% vs. −17.2 ± 2.2%, *p* < 0.01) compared to men. 

Both GLSEndo and GLSMyo were positively related with LV wall thickness (*p* < 0.001) and systolic diameter (*p* < 0.001) and negatively related to age, LV mass index and LVEF (*p* < 0.01). Conversely, GLSEpi was only negatively related to age and LV mass index (*p* < 0.05).

### 3.3. Segmental Longitudinal Strain

A total of 3600 segments were analyzed. The exclusion rate due to suboptimal tracking was 1.5%, yielding an overall feasibility of 98.5%. Unsuitable tracking was more frequent in apical segments and basal posterior.

A base to apex gradient was noted (*p* < 0.01) (Table 3).

**Figure 2:**
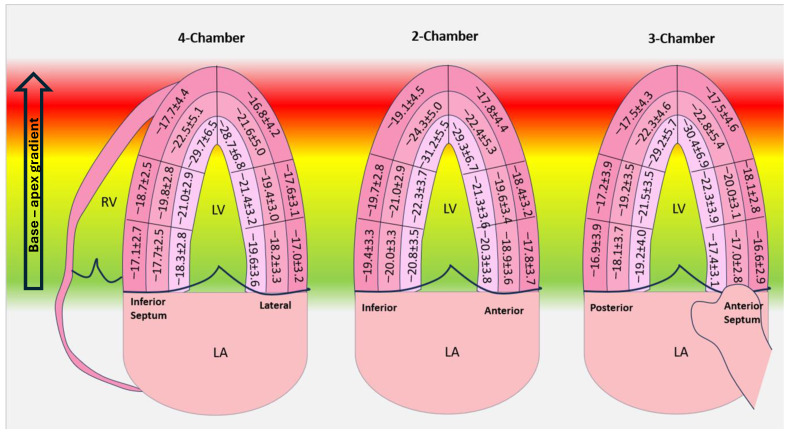
Average segmental strain values for subendocardial, mid-myocardial and epicardial longitudinal strain.

The average values and range for segmental strain are summarized in Table 4 and Figure 2. Overall, the segmental values ranged from −10% to −50% for endocardial LS, from −10% to −40% for mid-myocardial LS and from −8% to −31% for epicardial strain.

The average LS per walls was similar except for the inferior wall, which showed significantly higher values (*p* < 0.01) (Table 5).

The RASP was 0.73 ± 0.2 (range 0.3 to 1.3) for endocardial LS, 0.60 ± 0.1 (range 0.3 to 1.2) for mid-myocardial LS and 0.51 ± 0.1 (range 0.2 to 1.1) for epicardial LS, respectively.

In 10% of the subjects, the endocardial RASP was above 1, while the percentage was lower: (6%) for the mid-myocardial layer.

### 3.4. Reproducibility of the Measurements

The intra-observer reproducibility was excellent for GLS measurements. ICC coefficients were 0.95 (0.76–0.99, 95% CI) for GLSEndo, 0.92 (0.60–0.98, 95% CI) for GLSMyo and 0.91 (0.60–0.98, 95% CI) for GLSEpi.

The intra-observer average difference for segmental longitudinal strain ranged from 0.9 ± 0.8% (basal inferior, best reproducibility) to 4.8 ± 5.5% (apical inferior septum, poorest reproducibility). When assessed per levels, the highest variability was observed in the endocardial apical segments (3.7 ± 0.8%). Basal and mid segments showed similar variability for all layers (*p* > 0.5) and lower compared to that of the apical segments (*p* < 0.01).

## 4. Discussion

The myocardium has a unique and intricate architecture that contributes to its mechanical properties and specific functional requirements. The three myocardial layers, inner (endocardial), middle (universally accepted as mid-myocardial) and outer (epicardial), show distinct fiber orientation that leads to a structural anisotropy and spatially inhomogeneous electrical and mechanical properties.

The endocardial layer occupies approximatively a quarter of the LV wall thickness and consists mostly of longitudinal oriented fibers [12]. The fiber arrangement and the larger endocardial curvature in the context of an incompressible myocardial volume were proposed as explanations for the higher deformation during systole [13]. Moreover, the endocardial layer was shown to be more load dependent compared to the other layers [13]. The mid-myocardial layer occupies roughly two quarters of the thickness and consists of circumferentially arranged fibers while the outer layer is the thinnest and in contact with the pericardium and shows the lowest longitudinal deformation values.

Unlike traditional echocardiography, speckle tracking allows the quantification of multidirectional deformation and is highly sensitive and precise to subtle changes in myocardial motion, making it a valuable tool for the early detection of cardiac dysfunction [14,15,16]. Moreover, the ability to analyze deformation for each myocardium layer makes STE uniquely useful in certain clinical scenarios such as the early stages of ischemic injury that often develop firstly in the endocardium [17,18].

Although GLS has become a well-established parameter for assessing left ventricular function in daily practice, segmental LS values are integrated far less frequently into routine examination by clinicians. The absence of normograms derived from large cohorts, the high reported variability of LS and the inconsistency of values between vendors [8] are important factors that limit the widespread use of segmental LS.

Moreover, when interpreting segmental LS values, clinicians need to be aware of the normal range of values for each LV segment and layer, considering that deviations from these reference values could indicate the presence of myocardial pathology.

In the current study, we aimed to provide normal ranges for global and segmental layered LV deformation parameters derived from 2D-STE in a healthy population. Although a previous larger scale study reported segmental strain values in a healthy cohort [19], the results were extracted from tissue Doppler images using customized semi-automatic software, which limits their use in clinical practice. For this analysis, we opted to use the commercially available software that is predominantly used in the literature, facilitating the use of our results.

### 4.1. Global Longitudinal Strain

GLS showed an endocardial to epicardial strain gradient, with endocardial strain displaying the highest deformation. This is in accordance with previous reports [13,20,21].

In our study, the GLSEndo/GLSMyo ratio was 1.1, while the GLSEndo/GLSEpi was higher at 1.3, respectively. The ratio was not influenced by age or BSA.

While several mechanisms, such as the geometry of the LV, the myocardial fiber arrangement, the higher endocardial wall stress [13] and differences in echo intensities [22], were shown to play a role in the variances observed between the layers, an interesting large-scale study also suggested a certain heritability of the GLSEndo/GLSEpi gradient [23]. It remains to be explored whether the ratio could hold any significant diagnostic or prognostic value in clinical practice.

Both age and gender impacted the strain values, with females showing significantly higher values of GLS for all layers compared to men. Our results are supported by larger studies [19,24,25] that showed similar results.

LV geometry was shown to significantly impact GLS, and increased LV mass and concentric hypertrophy were shown to be associated with impaired GLS [26]. In our study, all three layers were negatively associated with LV mass, whereas only GLSEndo and GLSMyo were also associated with the LV end-systolic diameter. Interestingly, in our study population, GLS was not influenced by LA volumes.

### 4.2. Segmental Longitudinal Strain

Feasibility was excellent and significantly higher than that reported in the literature [27]. This is undoubtedly related to the biased selection of subjects with optimal image quality and thus may not reflect the reality of the measurement’s feasibility. However, in order to provide normal reference for strain values, we chose to include only subjects with good acoustic window.

Apical segments showed the highest variability in our study. This should be considered in clinical practice and may be due to certain features inherent to the tracking software (the more ample motion of the apical segments can lead to poorer tracking) or small changes in LV geometry during image acquisition that may have a higher impact on the apical segments and increase variability.

Basal segments demonstrated significantly lower values of LS for all layers compared to mid and apical segments. Anisotropy of the muscle fibers orientation, vicinity to the crux cordis, and the more pronounced curvature of the basal segments thus rendering a more perpendicular orientation of the ultrasound beam or differences in the angle of insonation [28] may all contribute to the lower values observed.

Of note, segments with LS values as low as −10% for endocardial and myocardial layers and −7% for epicardial were identified. Knowing the lower limit of LS for each segment and layer is significantly important in clinical practice due to the potential overlap of LS values between normal and infarcted or abnormal segments [29]. However, we could state that in the normal population, the lower LS values were identified predominantly in the basal segments.

When assessed per LV walls, we could not find any significant difference except for the inferior wall, which showed higher longitudinal strain.

Apical sparing defined as preserved LS in the apical segments despite impaired strain in the basal and mid segments demonstrated high accuracy in differentiating cardiac amyloidosis from other causes of cardiac hypertrophy [3,30,31] and infiltrative disease [32]. While a RASP above 1 was shown to accurately identify cardiac amyloidosis [3], several studies suggested that the pattern may not be entirely specific for cardiac amyloidosis [31,33]. Of note, in 10% of the subjects, the endocardial RASP was above 1, implying once again that further studies are warranted to validate the clinical utility of the parameter in the screening and diagnosis of cardiac amyloidosis.

Regarding the variability, it was higher in the apical segments, possibly related to the more pronounced curvature of the apex.

### 4.3. Study Limitation

First, the study was single-center and the homogenic ethnic characteristics (all subjects were Caucasians) may limit the extrapolation of our results to other ethnic groups.

Secondly, our population had a rather narrow range of age (18 to 55 years); therefore, the normal values provided in this study may not be accurate in subjects with older age. We opted to limit the maximum age at 55 years old considering the higher prevalence of cardiovascular risk factors above this age and the higher possibility for subclinical disease.

Last, the analysis was performed with a vendor-dependent software (EchoPac v204). The impact of the vendor-specific software on the measurements is significant and was demonstrated in multiple studies [8,27]. The results of this study are vendor specific and should not be extrapolated when the analysis is performed with other vendor-dependent or vendor-independent software.

## 5. Conclusions

The findings of this study provide data regarding the lower limit of normality of LS for each LV segment and suggest, for practical considerations, that an LS value below 10% should be considered abnormal in any segment. Further larger studies are warranted to confirm these findings.

## Figures and Tables

**Table 1:** Anthropometric and echocardiographic measurements of the study population.

Parameter	Unit	Mean ± STDEV
Age	(years)	37 ± 11
Gender, males/females	(%)	65/35
BSA	(m^2^)	1.8 ± 0.2
BMI	(kg/m^2^)	23 ± 4
Heart Rate	(bpm)	81 ± 14
SBP	(mmHg)	118 ± 9
DBP	(mmHg)	68 ± 7
IVSd	(mm)	8.4 ± 1.6
PWd	(mm)	8.3 ± 1.5
LVMi	(g/m^2^)	68 ± 15
LV EDVi	(mL/m^2^)	50 ± 11
LV ESVi	(mL/m^2^)	20 ± 5
LV SVi	(mL/m^2^)	32 ± 9
LV EF	(%)	59 ± 5
LV E wave	(cm/s)	83 ± 17
LV Edt	(ms)	157 ± 28
LV E/A		1.6 ± 0.5
LV s	(cm/s)	10 ± 2
LV E’	(cm/s)	16 ± 4
LV E/E’		5.3 ± 1
LA ESVi	(mL/m^2^)	22 ± 6
TAPSE	(cm)	23 ± 3
RV-EDAi	(cm/m^2^)	9 ± 2
RV-ESAi	(cm/m^2^)	5 ± 1
FAS	(%)	45 ± 9

BMI: body mass index; BSA: body surface area; DBP: diastolic blood pressure; EDAi: end-diastolic area indexed; EDVi: end-diastolic volume index; EF: ejection fraction; E/A: transmitral peak E/A ratio; E/E’: peak transmitral Ep/tissue Doppler E’ wave ratio; E: pulsed Doppler transmitral peak early diastolic wave; ESAi: end-systolic area indexed; ESVi: end-systolic volume index; FAS: fractional area shortening; IVSd: interventricular septum thickness, diastole; LV: left ventricular; LVMi left ventricular mass index; LA: left atrial; PWd: postero-lateral wall thickness, diastole; s: tissue Doppler peak systolic wave; SBP: systolic blood pressure, RV, right ventricle, SVi: stroke volume indexed, TAPSE: tricuspid annular plane systolic excursion.

**Table 2:** Anthropometric and echocardiographic measurements of the study population compared by gender.

Parameter	Unit	Males (*n* = 130)	Females (*n* = 70)
Age	(years)	35 ± 11	37 ± 12
BSA	(m^2^)	1.9 ± 0.2	1.6 ± 0.2 *
BMI	(kg/m^2^)	23 ± 4	21 ± 3 *
Heart Rate	(bpm)	79 ± 13	83 ± 14
SBP	(mmHg)	119 ± 9	116 ± 9
DBP	(mmHg)	68 ± 7	68 ± 8
IVSd	(mm)	9.0 ± 1.6	8.0 ± 1.5 *
PWd	(mm)	9.0 ± 1.5	8.0 ± 1.8 *
LVMi	(g/m^2^)	72 ± 14	61 ± 15 *
LV EDVi	(mL/m^2^)	52 ± 11	50 ± 11
LV ESVi	(mL/m^2^)	18 ± 4	21 ± 6 *
LV SVi	(mL/m^2^)	31 ± 8	32 ± 10
LV EF	(%)	59 ± 5	59 ± 5
LV E wave	(cm/s)	85 ± 16	81 ± 18
LV Edt	(ms)	156 ± 27	159 ± 28
LV E/A		1.6 ± 0.5	1.6 ± 0.5
LV s	(cm/s)	9 ± 2	10 ± 2
LV E’	(cm/s)	16 ± 3	15 ± 3
LV E/E’		5.5 ± 1	5.2 ± 1
LA ESVi	(mL/m^2^)	21 ± 6	22 ± 6
TAPSE	(cm)	22 ± 3	22 ± 3
RV-EDAi	(cm/m^2^)	9 ± 2	9 ± 2
RV-ESAi	(cm/m^2^)	5 ± 1	5 ± 1
FAS	(%)	46 ± 7	44 ± 9

*, *p* < 0.01, males compared to females; BMI: body mass index; BSA: body surface area; DBP: diastolic blood pressure; EDAi: end-diastolic area indexed; EDVi: end-diastolic volume index; EF: ejection fraction; E/A: transmitral peak E/A ratio; E/E’: peak transmitral Ep/tissue Doppler E’ wave ratio; E: pulsed Doppler transmitral peak early diastolic wave; ESAi: end-systolic area indexed; ESVi: end-systolic volume index; FAS: fractional area shortening; IVSd: interventricular septum thickness, diastole; LV: left ventricular; LVMi left ventricular mass index; LA: left atrial; PWd: postero-lateral wall thickness, diastole; s: tissue Doppler peak systolic wave; SBP: systolic blood pressure, RV, right ventricle, SVi: stroke volume indexed, TAPSE: tricuspid annular plane systolic excursion.

**Table 3:** Reference ranges for basal, mid and apical segments’ longitudinal strain.

Layer	Basal	Mid	Apical
Endocardial	−19.3 ± 3.6	−21.6 ± 3.5 *	−29.8 ± 6.4 *^,§^
Mid-myocardial	−18.3 ± 3.3	−19.9 ± 3.2 *	−22.7 ± 5.1 *^,§^
Epicardial	−17.5 ± 3.4	−18.3 ± 3.1 *	−17.7 ± 4.4 *^,§^

*, *p* < 0.01 compared to endocardial longitudinal strain; ^§^, *p* < 0.01 compared to mid-myocardial longitudinal strain.

**Table 4:** Average longitudinal strain values for endocardial, myocardial and epicardial layers per segments.

	Endocardial		Mid-Myocardial		Epicardial	
Basal	Mean ± SD	(Max, Min)	Mean ± SD	(Max, Min)	Mean ± SD	(Max, Min)
Anterior	−20.3 ± 3.8	(−30, −11)	−18.9 ± 3.7	(−28, −10)	−17.8 ± 3.7	(−28, −9)
Anterior septum	−17.4 ± 3.1	(−26, −10)	−17.0 ± 2.8	(−25, −10)	−16.6 ± 2.9	(−25, −9)
Inferior septum	−18.3 ± 2.8	(−25, −12)	−17.7 ± 2.5	(−23, −11)	−17.1 ± 2.7	(−24, −10)
Inferior	−20.8 ± 3.5	(−30, −12)	−20.0 ± 3.3	(−28, −11)	−19.4 ± 3.3	(−28, −10)
Posterior	−19.2 ± 4.0	(−30, −10)	−18.0 ± 3.7	(−28, −10)	−16.9 ± 3.9	(−27, −9)
Lateral	−19.6 ± 3.5	(−28, −11)	−18.2 ± 3.3	(−26, −10)	−17.0 ± 3.2	(−25, −8)
Mid						
Anterior	−21.3 ± 3.6	(−32, −12)	−19.6 ± 3.4	(−29, −10)	−18.4 ± 3.2	(−28, −10)
Anterior septum	−22.3 ± 3.9	(−34, −14)	−20.0 ± 3.1	(−30, −14)	−18.1 ± 2.8	(−27, −8)
Inferior septum	−21.0 ± 2.9	(−28, −15)	−19.8 ± 2.8	(−35, −14)	−18.7 ± 2.5	(−28, −12)
Inferior	−22.3 ± 3.7	(−32, −12)	−21.1 ± 2.9	(−29, −14)	−19.7 ± 2.8	(−28, −12)
Posterior	−21.5 ± 3.5	(−32, −13)	−19.2 ± 3.5	(−30, −10)	−17.2 ± 3.9	(−27, −8)
Lateral	−21.4 ± 3.2	(−30, −13)	−19.4 ± 3.0	(−26, −12)	−17.6 ± 3.1	(−25, −10)
Apical						
Anterior	−29.9 ± 6.7	(−46, −14)	−22.4 ± 5.3	(−36, −10)	−17.8 ± 4.4	(−29, −9)
Anterior septum	−30.4 ± 6.9	(−50, −17)	−22.8 ± 5.4	(−40, −12)	−17.5 ± 4.6	(−31, −8)
Inferior septum	−29.17 ± 6.5	(−47, −15)	−22.5 ± 5.1	(−35, −11)	−17.7 ± 4.4	(−31, −8)
Inferior	−31.1 ± 5.5	(−45, −16)	−24.3 ± 5.0	(−39, −12)	−19.1 ± 4.5	(−30, −8)
Posterior	−29.2 ± 5.7	(−46, −15)	−22.3 ± 4.6	(−36, −12)	−17.5 ± 4.3	(−30, −8)
Lateral	−28.7 ± 6.8	(−46, −15)	−21.6 ± 5.0	(−35, −12)	−16.8 ± 4.2	(−28, −8)

Values are expressed as average ± standard deviation.

**Table 5:** Average longitudinal strain values for endocardial, myocardial and epicardial layers per walls.

	Endocardial	Mid-Myocardial	Epicardial
	Mean ± SD	Mean ± SD	Mean ± SD
Inferior septum	−23.0 ± 6.6	−20.0 ± 4.2	−17.9 ± 3.4
Lateral	−23.2 ± 6.2	−19.7 ± 4.1	−17.1 ± 3.6
Inferior	−24.8 ± 6.3	−21.8 ± 4.2	−19.4 ± 3.6
Anterior	−23.6 ± 6.3	−20.3 ± 4.4	−18.0 ± 3.8
Posterior	−23.4 ± 6.2	−19.9 ± 4.4	−17.2 ± 4.0
Anterior septum	−23.4 ± 7.3	−19.9 ± 4.6	−17.4 ± 3.6

Values are expressed as average ± standard deviation.

## Data Availability

Data are available upon request in specific conditions.

## References

[1] Voigt J.U., Pedrizzetti G., Lysyansky P., Marwick T.H., Houle H., Baumann R., Pedri S., Ito Y., Abe Y., Metz S. (2015). Definitions for a common standard for 2D speckle tracking echocardiography: Consensus document of the EACVI/ASE/Industry Task Force to standardize deformation imaging. J. Am. Soc. Echocardiogr..

[2] Kalam K., Otahal P., Marwick T.H. (2014). Prognostic implications of global LV dysfunction: A systematic review and meta-analysis of global longitudinal strain and ejection fraction. Heart.

[3] Phelan D., Collier P., Thavendiranathan P., Popović Z.B., Hanna M., Plana J.C., Marwick T.H., Thomas J.D. (2012). Relative apical sparing of longitudinal strain using two-dimensional speckle-tracking echocardiography is both sensitive and specific for the diagnosis of cardiac amyloidosis. Heart.

[4] Risum N., Jons C., Olsen N.T., Fritz-Hansen T., Bruun N.E., Hojgaard M.V., Valeur N., Kronborg M.B., Kisslo J., Sogaard P. (2012). Simple regional strain pattern analysis to predict response to cardiac resynchronization therapy: Rationale, initial results, and advantages. Am. Heart J..

[5] Edvardsen T., Skulstad H., Aakhus S., Urheim S., Ihlen H. (2001). Regional myocardial systolic function during acute myocardial ischemia assessed by strain Doppler echocardiography. J. Am. Coll. Cardiol..

[6] Uppu S.C., Shah A., Weigand J., Nielsen J.C., Ko H.H., Parness I.A., Srivastava S. (2015). Two-Dimensional Speckle-Tracking-Derived Segmental Peak Systolic Longitudinal Strain Identifies Regional Myocardial Involvement in Patients with Myocarditis and Normal Global Left Ventricular Systolic Function. Pediatr. Cardiol..

[7] Sperlongano S., D’Amato A., Tagliamonte E., Russo V., Desiderio A., Ilardi F., Muscogiuri G., Esposito G., Pontone G., Esposito G. (2022). Acute myocarditis: Prognostic role of speckle tracking echocardiography and comparison with cardiac magnetic resonance features. Heart Vessel..

[8] Mirea O., Pagourelias E., Duchenne J., Bogaert J., Thomas J.D., Badano L.P., Voigt J.-U., on behalf of the EACVI-ASE-Industry Standardization Task Force (2018). Intervendor Differences in the Accuracy of Detecting Regional Functional Abnormalities: A Report From the EACVI-ASE Strain Standardization Task Force. J. Am. Coll. Cardiol. Imaging.

[9] Lang R.M., Badano L.P., Mor-Avi V., Afilalo J., Armstrong A., Ernande L., Flachskampf F.A., Foster E., Goldstein S.A., Kuznetsova T. (2015). Recommendations for cardiac chamber quantification by echocardiography in adults: An update from the American Society of Echocardiography and the European Association of Cardiovascular Imaging. Eur. Heart. J. Cardiovasc. Imaging.

[10] Liao J.J., Capen R.C., Schofield T.L. (2006). Assessing the reproducibility of an analytical method. J. Chromatogr. Sci..

[11] Nagueh S.F., Smiseth O.A., Appleton C.P., Byrd B.F., Dokainish H., Edvardsen T., Flachskampf F.A., Gillebert T.C., Klein A.L., Lancellotti P. (2016). Recommendations for the Evaluation of Left Ventricular Diastolic Function by Echocardiography: An Update from the American Society of Echocardiography and the European Association of Cardiovascular Imaging. Eur. Heart J. Cardiovasc. Imaging.

[12] Ho S.Y. (2009). Anatomy and myoarchitecture of the left ventricular wall in normal and in disease. Eur. J. Echocardiogr..

[13] Smiseth O.A., Torp H., Opdahl A., Haugaa K.H., Urheim S. (2016). Myocardial strain imaging: How useful is it in clinical decision making?. Eur. Heart J..

[14] Edvardsen T., Helle-Valle T., Smiseth O.A. (2006). Systolic Dysfunction in Heart Failure with Normal Ejection Fraction: Speckle-Tracking Echocardiography. Prog. Cardiovasc. Dis..

[15] Ewe S.H., Haeck M.L., Ng A.C., Witkowski T.G., Auger D., Leong D.P., Abate E., Marsan N.A., Holman E.R., Schalij M.J. (2015). Detection of subtle left ventricular systolic dysfunction in patients with significant aortic regurgitation and preserved left ventricular ejection fraction: Speckle tracking echocardiographic analysis. Eur. Heart J. Cardiovasc. Imaging.

[16] Nakai H., Takeuchi M., Nishikage T., Lang R.M., Otsuji Y. (2009). Subclinical left ventricular dysfunction in asymptomatic diabetic patients assessed by two-dimensional speckle tracking echocardiography: Correlation with diabetic duration. Eur. J. Echocardiogr..

[17] Ono S., Waldman L.K., Yamashita H., Covell J.W., Ross J. (1995). Effects of coronary artery reperfusion on transmural myocardial remodeling in dogs. Circulation.

[18] Altiok E., Neizel M., Tiemann S., Krass V., Kuhr K., Becker M., Zwicker C., Koos R., Lehmacher W., Kelm M. (2012). Quantitative analysis of endocardial and epicardial left ventricular myocardial deformation-comparison of strain-encoded cardiac magnetic resonance imaging with two-dimensional speckle-tracking echocardiography. J. Am. Soc. Echocardiogr..

[19] Dalen H., Thorstensen A., Aase S.A., Ingul C.B., Torp H., Vatten L.J., Stoylen A. (2010). Segmental and global longitudinal strain and strain rate based on echocardiography of 1266 healthy individuals: The HUNT study in Norway. Eur. J. Echocardiogr..

[20] Shi J., Pan C., Kong D., Cheng L., Shu X. (2016). Left Ventricular Longitudinal and Circumferential Layer-Specific Myocardial Strains and Their Determinants in Healthy Subjects. Echocardiography.

[21] Alcidi G.M., Esposito R., Evola V., Santoro C., Lembo M., Sorrentino R., Lo Iudice F., Borgia F., Novo G., Trimarco B. (2018). Normal reference values of multilayer longitudinal strain according to age decades in a healthy population: A single-centre experience. Eur. Heart J. Cardiovasc. Imaging.

[22] Aygen M., Popp R.L. (1987). Influence of the orientation of myocardial fibers on echocardiographic images. Am. J. Cardiol..

[23] Huttin O., Xhaard C., Dandine-Roulland C., Le Floch E., Bacq-Daian D., Lamiral Z., Bozec E., Deleuze J.-F., Zannad F., Rossignol P. (2023). Layer myocardial strain is the most heritable echocardiographic trait. Eur. Heart J. Cardiovasc. Imaging.

[24] Sugimoto T., Dulgheru R., Bernard A., Ilardi F., Contu L., Addetia K., Caballero L., Akhaladze N., Athanassopoulos G.D., Barone D. (2017). Echocardiographic reference ranges for normal left ventricular 2D strain: Results from the EACVI NORRE study. Eur. Heart J. Cardiovasc. Imaging.

[25] Nyberg J., Jakobsen E.O., Østvik A., Holte E., Stølen S., Lovstakken L., Grenne B., Dalen H. (2023). Echocardiographic Reference Ranges of Global Longitudinal Strain for All Cardiac Chambers Using Guideline-Directed Dedicated Views. JACC Cardiovasc. Imaging.

[26] Anan R., Imoto T., Onizuka K., Watanabe H., Mori W., Murakoso M. (2023). Concentric hypertrophy geometry is a significant determinant of impaired global longitudinal strain in patients with normal cardiac structure and function. Heliyon.

[27] Mirea O., Pagourelias E.D., Duchenne J., Bogaert J., Thomas J.D., Badano L.P., Voigt J.U. (2018). EACVI-ASE-Industry Standardization Task Force. Variability and Reproducibility of Segmental Longitudinal Strain Measurement: A Report From the EACVI-ASE Strain Standardization Task Force. JACC Cardiovasc. Imaging.

[28] Forsha D., Risum N., Rajagopal S., Dolgner S., Hornik C., Barnhart H., Kisslo J., Barker P. (2015). The influence of angle of insonation and target depth on speckle-tracking strain. J. Am. Soc. Echocardiogr..

[29] Bière L., Donal E., Terrien G., Kervio G., Willoteaux S., Furber A., Prunier F. (2014). Longitudinal strain is a marker of microvascular obstruction and infarct size in patients with acute ST-segment elevation myocardial infarction. PLoS ONE.

[30] Nardozza M., Chiodi E., Mele D. (2017). Left Ventricle Relative Apical Sparing in Cardiac Amyloidosis. J. Cardiovasc. Echogr..

[31] Wali E., Gruca M., Singulane C., Cotella J., Guile B., Johnson R., Mor-Avi V., Addetia K., Lang R.M. (2023). How Often Does Apical Sparing of Longitudinal Strain Indicate the Presence of Cardiac Amyloidosis?. Am. J. Cardiol..

[32] Lagies R., Beck B.B., Hoppe B., Sreeram N., Udink Ten Cate F.E. (2013). Apical sparing of longitudinal strain, left ventricular rotational abnormalities, and short-axis dysfunction in primary hyperoxaluria type 1. Circ. Heart Fail..

[33] Abecasis J., Lopes P., Santos R.R., Maltês S., Guerreiro S., Ferreira A., Freitas P., Ribeiras R., Andrade M.J., Manso R.T. (2023). Prevalence and significance of relative apical sparing in aortic stenosis: Insights from an echo and cardiovascular magnetic resonance study of patients referred for surgical aortic valve replacement. Eur. Heart J. Cardiovasc. Imaging.

